# Sustainable Membrane Development: A Biopolymer Approach

**DOI:** 10.3390/polym17243260

**Published:** 2025-12-08

**Authors:** Mónica Morales-Jiménez, Gabino A. Martínez-Gutiérrez, Eduardo Perez-Tijerina, Francisco Solis-Pomar, Manuel F. Meléndrez, Daniel A. Palacio

**Affiliations:** 1 Centro Interdisciplinario de Investigación para el Desarrollo Integral Regional, Unidad Oaxaca, Instituto Politécnico Nacional, Calle Hornos 1003, Colonia Noche Buena, Santa Cruz Xoxocotlán, Oaxaca City 71230, Mexico; mmoralesj@ipn.mx (M.M.-J.); gamartinezg@ipn.mx (G.A.M.-G.); 2 Laboratorio de Nanociencias y Nanotecnología, Facultad de Ciencias Físico Matemáticas (CICFM-FCFM), Universidad Autónoma de Nuevo León (UANL), San Nicolás de los Garza 66451, Mexico; 3 Facultad de Ciencias de la Rehabilitación y el Calidad de Vida, Universidad San Sebastián Campus Las Tres Pascualas, Lientur 1439, Concepción 4030000, Chile; 4 Biobased and Bioinspired Biomaterials Research Group, Laboratory of Functional Polymers and Environment, Departamento de Polímeros, Facultad de Ciencias Químicas, Universidad de Concepción, Edmundo Larenas 129, Casilla 160-C, Concepción 4070371, Chile

**Keywords:** membrane technology, biopolymer, green membrane, sustainability

## Abstract

Sustainable membranes for efficient separation processes are increasingly necessary to counteract the significant environmental and human health impacts of manufacturing conventional membranes, which rely on synthetic polymers, toxic solvents, and harmful additives. A greener approach currently involves the use of bio-based polymers, blending synthetic polymers with biopolymers, utilizing nanocomposites, and greener solvents. Biopolymers are emerging as an environmentally friendly alternative for developing polymeric membranes due to their biological, biodegradable, recyclable, and biocompatible properties. However, the development of sustainable biopolymer-based membranes poses greater challenges to achieving a truly low environmental impact across all aspects of raw material production, manufacturing methods, operational systems, and waste disposal. Another challenge for its market competitiveness is achieving high functional and operational performance, wider applications, low commercial costs, and strong scale-up potential. This critical review assesses the current state of sustainability in membrane manufacturing based on recent literature. It also evaluates the role of biopolymers in sustainable membranes and discusses research opportunities in biopolymer production for membrane manufacturing, highlighting biotechnological tools, the circular economy and waste valorization.

## 1. Introduction

Membrane science and technology have emerged as promising alternatives for separation techniques, offering high quality, selectivity, and stability. Different types and configurations of membranes have been developed using various methods, including phase inversion, interfacial polymerization, stretching, and electrospinning. Membranes can be classified as solid or liquid and can take on different designs, including plate-and-frame, hollow fiber, tubular, and spiral-wound configurations [1]. Membrane applications are diverse and include water treatment, gas purification, biomedical uses, pharmaceuticals, biotechnology, chemical processing, aerospace, petrochemicals, food packaging, clarification, concentration, metallurgical processes, power generation, and the microelectronics industry [2].

Despite the high efficiency of membrane separation processes, membrane technology faces several challenges, including limited membrane lifetime, low selectivity for specific applications, imprecise ion and molecule separations, low solute-solvent separation, membrane contamination, and high capital and operational costs, flux and quality loss due to membrane fouling and concentration polarization [3,4]. Membrane fouling is a significant challenge to membrane separation performance, and monitoring it is a complex process. Undesired materials (e.g., particles, organic and inorganic compounds, and microorganisms) deposit and accumulate on the membrane surface, causing reversible or irreversible damage, flux decline, deterioration of selectivity and permeability, and reduced lifespan [5]. Fouling mitigation strategies include membrane disinfection, optimizing operational conditions, monitoring fouling, predicting fouling, developing membrane cleaning and maintenance protocols, membrane surface modifications and functionalization, feed spacers, and the development of new fouling-resistant membranes [6,7]. Layer-by-layer surface coating [8], slip casting, cation complexation, radical grafting, and polymerization methods using nanoparticles [9], zwitterionic materials, copolymers, graphene oxide, and carbon nanotubes have been explored for the fabrication of fouling-resistant membranes. All these attempts have been successful; however, the compounds and materials used are mostly toxic and expensive, and most environmentally friendly techniques need further research [10].

Additionally, membrane manufacturing has significant environmental impacts due to the discharge of highly toxic and non-recyclable effluents. These issues contribute to severe global warming, terrestrial acidification, eutrophication, marine ecotoxicity, and both carcinogenic and non-carcinogenic toxicity to humans, as well as concerns related to land use and fossil resource scarcity [11,12]. This situation presents a significant sustainability challenge that necessitates reducing the adverse effects associated with membrane manufacturing. To address this, there is a growing push for the development of sustainable membranes that are both cost-effective and environmentally friendly. This development focuses on bio-based polymers, including biopolymers and new metabolite polymers derived from biomass-originated monomers, such as bio-poly(trimethylene terephthalate). It also encompasses conventional petrochemical polymers made from bio-derived substances such as bio-polyethylene and bio-polypropylene. Furthermore, the use of green solvents, such as Cirene, lactic acid esters, PolarClean, triacetin, dimethyl sulfoxide, and green additives is emphasized [13].

Within a circular economy framework, the biological and renewable origin of a polymer, along with its biodegradability, are crucial criteria for membrane technology, making it a significant contribution to sustainability. Biopolymers are a selected group of polymers that mostly meet these criteria and are derived from living matter [14]. Biopolymers are commonly obtained from agro-industrial waste, including sugarcane bagasse, crustaceans, sisal fibers, wheat straw, coffee husk, banana peel, wood pulp, rice husk, as well as from plants, seaweed, pigs, cows, and fish. They can also be produced through fermentation using microorganisms [15]. These agro-industrial sources may appear harmless; however, the extraction methods for biopolymers typically involve acid or alkali treatments, which can result in extensive processing with significant environmental and economic impacts [11]. For instance, when extracting chitin for film preparation, using mild ball milling to produce protein-rich chitin pulp results in a lower ecological footprint compared to traditional chitin nanocrystal processes [16]. Through a biorefinery approach, chitin and chitosan obtained from lobster and spider crab shells can also be used for the preparation of nanocomposite films, which can also help reduce carbon dioxide emissions [17].

Today, biopolymers are a promising alternative for biomaterials development, environmentally friendly and non-toxic. Although biopolymer-based membranes cannot yet replace petroleum-based membranes, and green manufacturing processes require further clarification, additional research is needed to improve efficiency, production processes, recycling protocols, and adequate waste disposal [18,19]. This review examines the current state of sustainable membrane manufacturing and its prospects, emphasising the importance of developing novel biopolymers with promising mechanical and resistance properties that can ever replace synthetic polymers in the fabrication of polymeric membranes.

## 2. Membrane Manufacturing Pollution

The membrane preparation process can produce over 50 billion liters of contaminated water annually. It is projected that between 23 and 37 million tons of plastic waste will enter water bodies by 2040. Additionally, around 31.9 million tons of plastic waste are deposited into the environment each year. The most common materials used for membrane production are polyvinylidene fluoride (PVDF) as the polymer and *N*-methyl-2-pyrrolidone (NMP) as the solvent. Unfortunately, these materials significantly contribute to environmental damage [11,20,21,22]. NMP is the most frequently used solvent for dialysis membrane production, which can remain in the final product. This compound is miscible with water and is not broken down by chemical hydrolysis, making it an undesirable solvent for the fabrication of a green membrane. However, its production for this purpose is approximately 2000 to 4000 tons per year [23].

Microplastics (e.g., polyvinylidene fluoride, polytetrafluoroethylene, fluorinated ethylene propylene, perfluoroalkoxy polymer, and polyvinyl fluoride), polycyclic aromatic hydrocarbons, polychlorinated biphenyls, bisphenol A, and radionuclides are substances originating from the manufacturing and breakdown of various materials, including synthetic membranes. These substances are recalcitrant compounds, meaning that their persistence in the environment is nearly infinite, leading to pollution in soil, air, wildlife, human tissues, and water bodies, including drinking water, groundwater, oceans, lakes, rivers, rainwater, snow, and municipal sewage [24]. Alarmingly, microplastics originating from membrane materials, additives, and polymeric components of the ultrafiltration membrane system have recently been documented in drinking water. Microplastics in the ultrafiltration permeate ranged from 694.1 to 1232.6 items/L. Also, the operational conditions and membrane cleaning can increase the amount of microplastics [25].

Microplastics and nanoplastics have been detected in the human heart, kidney, liver, brain, blood, and urine. This indicates that human exposure to these plastics has resulted in their entry into the human body via inhalation, ingestion of drinking water or food, and skin contact. There is even evidence suggesting a potential risk of exposure to plastic particles in patients undergoing dialysis. The global concern focuses on their toxic effects, including cytotoxicity, immune disruption, disturbance of energy homeostasis, and disrupted metabolism [26]. Airborne microplastics can be inhaled and become lodged in various tissues, causing obstructions, oxidative stress, and potentially leading to cancer. Fragments and fibers, ranging from white to transparent and blue, measuring 1–1000 μm, are among the most common types of airborne microplastics found in urban air [27]. A recent study found nine microplastics in high-altitude cloud water, identified within the atmospheric boundary layer and the free troposphere at altitudes ranging from 1300 to 3776 m. The researchers proposed that this microplastic may influence cloud formation and, consequently, contribute to climate change [28]. Nanoplastics also represent a potential risk to public health.

In contrast, nanomaterials and nanocomposites based on synthetic polymers have become a remarkable option for potential uses such as desalination membranes with high permeability and selectivity; however, their environmental impacts have been underestimated. Nanomaterials influence the environment through physical (e.g., deposition and agglomeration) and biological (e.g., bioaccumulation in plants, as well as in terrestrial and aquatic animals) mechanisms. Due to their small size (1–100 nm), large specific surface area, and high reactivity, nanoparticles can engage in redox reactions, dissolution, and photochemical reactions. Their toxicity may increase when they penetrate cell membranes and disrupt physiological processes [29].

## 3. Current Sustainable Membrane Manufacturing

Today, there is increasing interest in adopting greener alternatives for membrane preparation. Approaches include the use of bio-based polymers, blended synthetic polymers, and biopolymers [30]. Other options consist of nanocomposites, recycled polymer plastic waste, recycled membranes, greener solvents (e.g., water, ethanol, ionic liquids, supercritical carbon dioxide, 1-heptanol, ethylene glycol, 1-octanol, 1-butanol, 1-propanol, glycerol diacetate, isobutyl acetate, propylene carbonate, diethyl carbonate, and cyclopentanone), mixtures of toxic and environmentally friendly solvents, and natural additives. These natural additives may include deep eutectic solvents, clays, biochar, gum arabic, lignin, cellulose, and nanoparticles derived from leaf extracts [13,31,32,33].

Cellulose derivatives, which are bio-based polymers, are widely studied for membrane preparation. The chemical modification of cellulose aims to improve membrane performance. However, this process often involves chemicals and solvents that significantly harm the environment. To evaluate both the environmental and economic aspects of the materials used in membrane production, life cycle and techno-economic analyses are essential [33]. The global warming potential was evaluated through a life-cycle assessment of dissolved cellulose in common solvents used for cellulose fiber production. In total, 130.0–332.4 Kg CO_2_-eq/Kg fiber was detected for 1-butyl-3-methylimidazolium chloride solvent. 87.1 Kg CO_2_-eq/Kg fiber was detected for *N*-methylmorpholine-*N*-oxide solvent. Additionally, a 41.3–70 Kg CO_2_-eq/Kg fiber was detected for the alkali/urea solvent [34]. For the industrial scale of cellulose bleaching methods during nanocellulose fibers production, life-cycle analysis revealed 1036.71 Kg CO_2_-eq/Kg nanocellulose using NaClO_2_ treatment, 898.58 Kg CO_2_-eq/Kg nanocellulose using H_2_O_2_ treatment, and 975.33 Kg CO_2_-eq/Kg nanocellulose using H_2_O_2_-acetic acid treatment [35]. These analyses help assess the sustainability of replacing fossil materials with seemingly sustainable alternatives. Life cycle analysis is a valuable tool for determining the environmental impact of membrane production or a process throughout all its stages, providing information on critical environmental points and guiding the development of a strategic and sustainable infrastructure. When comparing cellulose-derived polymers to polyvinylidene fluoride (PVDF) and polysulfone (PSF), the reductions in environmental impact were minimal, particularly in terms of global warming potential and the risk of exposure to ionizing radiation. Regarding marine ecotoxicity, human carcinogenic potential, land use, and fossil resource scarcity, the ecological impacts of PVDF, PSF, and cellulose-derived polymers were found to be similar. Notably, cellulose-derived polymers showed a higher impact than PSF in terms of human non-carcinogenic toxicity potential and ionizing radiation potential [11].

A similar situation may occur with green solvents if their production causes a significant environmental impact. One of the primary challenges in utilizing green solvents for sustainable membrane fabrication is the limited solubility of specific polymers. Consequently, combining green and non-green solvents is often necessary. Additionally, membranes must be fabricated with competitive structures and selectivity. Cellulose-derived polymers have demonstrated adequate solubility in green solvents such as methyl lactate, ethyl lactate, dimethyl sulfoxide, and PolarClean [36]. Research indicates that the environmental impact of producing 1 kg of ethylene carbonate as a green solvent is considerably lower compared to solvents like NMP, dimethylformamide, and dimethylacetamide [15]. This suggests that organic carbonates could be promising alternatives to conventional green solvents. Ideally, the aim is to utilize a non-solvent membrane process and employ methods such as lithography, 3D printing, or environmentally friendly approaches for synthesizing green solvents [37]. Three-dimensional printing is revolutionizing biomaterials manufacturing as an advanced additive manufacturing technique that creates three-dimensional materials layer by layer, step by step, based on digital design. More ambitiously, 4D printing offers the additional dimension of time, allowing for changes in shape or function over time. Three- and four-dimensional printed materials could be designed to be recycled, reused, and composted [38]. In addition, 3D printing is emerging as a promising alternative for developing feed spacer membranes, offering a wide variety of geometries compared to a standard diamond-shaped spacer and reducing energy consumption in membrane filtration system by increasing flux and enhancing permeability. However, the remaining concerns are the materials used and the toxicity of volatile compounds during the 3D printed process, which involves polyamide, thermoplastic polyurethane, polypropylene, and acrylate monomers [39]. Further research is needed on environmentally friendly materials for feed spacer membranes.

**Figure 1:**
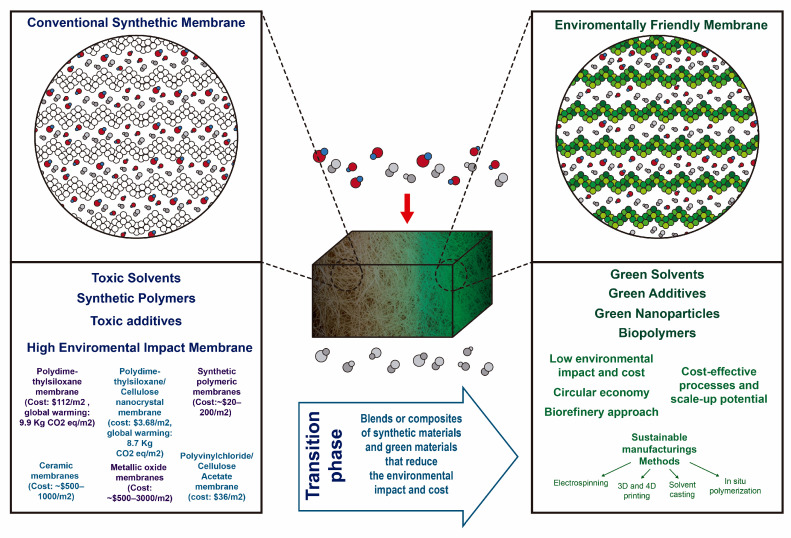
Schematic illustration of sustainable membrane development [44,45,46].

A truly sustainable solution has yet to be realized. All attempts described could significantly reduce the environmental impact of membrane manufacturing, with an emphasis on including the use of renewable energy sources [40,41]. Today, as most commercial membranes consist of synthetic polymer materials [42]. The transition towards sustainable membrane development [43] is ongoing, as illustrated in Figure 1.

**Figure 2:**
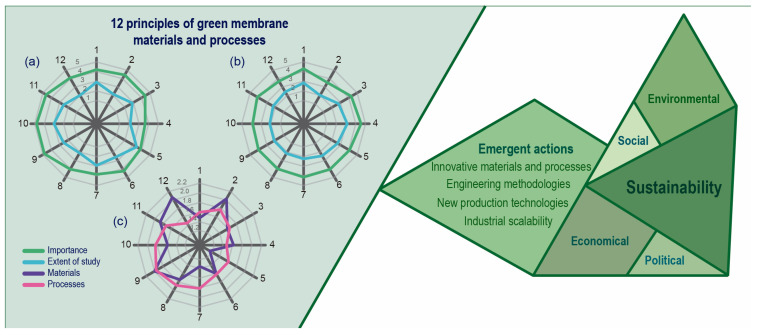
Study areas for a membrane-sustainable future. (**a**) Principles of green membrane materials, (**b**) principles of green membrane processes, and (**c**) principles of green membrane materials and processes that require more attention [47,55].

Recently, Szekely (2024) established 12 strategic principles for green membrane materials and 12 strategic methods to enhance the sustainability of membrane technology [47]. The principles for green membrane materials focus on a cradle-to-grave mindset, reducing wastewater generation, and ensuring the reproducibility of membrane materials. For membrane processes, concepts such as automation, process analytical technology (PAT) monitoring, and the use of artificial intelligence have been identified as more critical. The author suggests that a more comprehensive investigation of all these principles is necessary, as illustrated in Figure 2.

Convincingly, achieving membrane sustainability under a global approach will be strictly based on circular economy, biorefinery, and waste and wastewater valorization frameworks, which involve minimizing carbon and water footprint, reducing consumption of natural resources, using renewable energy, extending the membrane service life, reusing and recycling membranes, and high-added-value products recovering [48,49,50]. The useful life of synthetic membranes ranges from 3 to 7 years, and a minimal percentage of 9% of plastic waste is recycled [48,51]. Synthetic membrane recycling can minimize the environmental impact, considering a careful examination of pollutants and hazardous compounds retained in the membrane’s porous structure for processing new products or membranes. It will be crucial to redirect the recycled material to produce high (upcycling) or low-performance (downcycling) products [48]. Alkaline-NaClO treatment was explored for foulant (Si-Al) remotion of end-of-life reverse osmosis membranes, and the membranes were converted into functional downcycled nanofiltration membranes with water permeance of 27.0 L/m^2^h·bar, and ultrafiltration membranes with water permeance of 60–90 L/m^2^h·bar [52].

A significant challenge lies in ensuring sustainability during both the fabrication and operation of membranes, as well as in the development of promising functional materials that support a sustainable membrane technology. Notably, membrane separation will be one of the most effective solutions for pressing environmental issues, such as addressing water scarcity [53] and mitigating radon air pollution [54]. A scientific push in membrane separation processes is primarily aimed at ensuring the reliability of membrane industrial operations; the primary goal is to develop high-performance materials, robust production methods, and practical process engineering and design tools [55]. To envision a sustainable future for membrane technology, it is essential to promote synergistic research across emergent and orchestrated actions, all while emphasizing sustainability, as shown in Figure 2.

The principles of green membrane materials include 1. considering greener compounds, 2. reducing wastewater generation, 3. using less hazardous materials, 4. reducing the number of constituents, 5. using benign surface modifications, 6. reducing complexity and steps, 7. using ambient conditions, 8. reducing the raw material, 9. ensuring reproducibility, 10. designing for robust performance, 11. designing for scalability, and 12. having a cradle-to-grave mindset. The principles of green membrane processes include 1. reducing processing steps, 2. reducing buffer tanks and auxiliaries, 3. reducing solvent consumption, 4. reducing energy consumption, 5. increasing the concentration, 6. reducing footprint, 7. designing for safety, 8. monitoring with process analytical technology (PAT), 9. integrating automation, 10. exploiting artificial intelligence (AI), 11. promoting closed-loop systems, and 12. employing renewable energy sources.

Sustainability suggests a fair balance between human activities and needs and the environment, allowing for long-term coexistence. This concept is complex, considering the massive use of synthetic membrane materials. Kim et al. (2025) proposed six Sustainable Development Goals aligned with the 17 Sustainable Development Goals of the United Nations [56]. The goals are focused towards sustainable industrial processes and sustainable membrane manufacturing, such as precise separation, challenging separation, process intensification, sustainable operation, green membrane fabrication, and recycling and upcycling.

## 4. Biopolymers

The biopolymers are a set of polymers derived from plants, animals, and microorganisms, such as starch and its derivatives, cellulose and its derivatives, chitin, chitosan, hyaluronan, xanthan, pectin, inulin, lignin, arabic gum, guar gum, gum tragacanth, carrageenan, natural latex, elastin, gluten, albumin, tannin, ulvan, fucans, laminarin, alginate, fructans, agavins, casein, collagen, pullulan, dextrans, sulfated polysaccharides from microalgae, phycobiliproteins, polyglutamic acid, and polyhydroxyalkanoates [57]. Biopolymers also refer to polymers produced naturally from renewable materials, such as polylactic acid and polybutylene succinate. A considerable number of polymers are chemically synthesised and exhibit excellent biodegradability, which makes them considered biopolymers such as polyethylene succinate [58]. The definition of a biopolymer remains ambiguous; however, its main characteristics include biodegradability, biocompatibility, and sustainability.

Starch is a homopolymer of α-D-glucopyranose units, which consist of linear amylose and branched amylopectin. Its granules are semi-crystalline, and the crystallization degree ranges from 15 to 45%. Starch had poor solubility in cool water and organic solvents. Chemical, physical, and enzymatic modifications are frequently applied to starch to enhance its thermal stability, improve its rheological properties and solubility, decrease its viscosity, and decrease its swelling power and retrogradation [59]. Cellulose is a linear chain polymer of β(1–4) linked D-glucopyranose units from plant and bacterial sources. It is insoluble in water and most organic solvents, offering non-toxicity and strong tensile and compressive strength. Chemical modifications are applied to the cellulose structure to enhance solubility, mechanical properties, and functionality [60]. Chitin is a linear polymer of *N*-acetyl-D-glucosamine units, and exists in α, β and γ-chitin. It is insoluble in organic solvents and water. Through the deacetylation process, chitosan is obtained from chitin. Both biopolymers are non-toxic, biocompatible, and bioadhesive, and can form films [61]. Carrageenan is a sulfated polygalactan that consists of alternating units of D-galactose and 3,6-anhydrogalactose. It is a biopolymer derived from marine red algae, non-toxic, and soluble in water; for that reason, it is widely used as a thickening agent and stabilizer. According to the sulfation degree, carrageenan exists in three types: ϰ-carrageenan, ϊ-carrageenan, and λ-carrageenan. ϰ-carrageenan offers a higher gel melting temperature and forms strong, brittle, and rigid gels with high water retention [62]. Polyhydroxyalkanoates consist of hydroxyalkanoate monomers. Short chains (4–5 carbons) exhibit high melting points and high crystallinity, medium chains (6–14 carbons) offer more elastic properties, and long chains (>14 carbons) exhibit low glass transition temperatures and low tensile strength [63].

## 5. The Role of Biopolymers in Membrane Manufacturing

The primary goal for sustainable membrane preparation is to ensure membrane cleaner recyclability [32], biodegradability, bio-safety, bioactivity, and minimal environmental impact [64]. Biopolymers appear to be the most promising alternative for achieving these qualities in membrane preparation. Consequently, research has focused on developing sustainable, novel biopolymers and innovative production methods (such as cross-linking and co-polymerization) that are compatible with green solvents, as well as new strategies to enhance their properties and broaden their applications. Additionally, the incorporation of silver nanoparticles into polylactic acid membranes has been investigated to improve hydrophilicity and antibacterial properties [65].

Recent advancements in greener approaches have led to the development of porous membranes using a polylactic acid (PLA)-solvent system combined with eco-friendly solvents such as dimethyl sulfoxide (DMSO), methyl lactate, dimethyl isosorbide, γ-valerolactone, and Cyrene [66]. Kim et al. (2024) demonstrated the creation of solvent-resistant cellulose membranes using the green solvents methyl lactate and triethyl phosphate [67]. Although these membranes demonstrated resistance to strong aprotic solvents, their mechanical properties still do not match those of commercial membranes. Additionally, Yang et al. (2025) introduced a co-electrospinning–electrospray method as an environmentally friendly strategy to develop a multiscale fibrous filter [68]. This filter is composed of bacterial cellulose nanofibers along with micro- and submicron fibers made from polylactic acid, achieving an impressive removal efficiency of 99.68% for particulate matter measuring 0.3 μm. Moreover, an electro-blow spinning method has also been employed to produce polylactic acid nanofibers, which demonstrated filtration efficiency of up to 98% [69]. For separation processes, chitin and chitosan biopolymer-based membranes have increased rejection percentages and anti-fouling properties due to the dense nature of the resultant membrane [70]. Radoor et al. (2024) developed a hydrogel filtration membrane using calcium alginate and carrageenan to remove toxic cationic dye [71]. The membrane had good hydrophilicity, compatibility, and recyclability over nine cycles. A rejection rate of 100% was achieved at a concentration of 10 mg/L of methylene blue dye, a pressure of 0.1 MPa, a pH of 7, and a temperature of 25 °C.

Biopolymers-based composites and nanocomposites have emerged as an innovative tool to enhance the properties and functionalities of native biopolymers through the use of fillers [19]. A novel thin film was composed of chitosan, carboxymethyl cellulose, tannic acid, glycerol, and Tween 80; this bio-composite membrane exhibited a tensile strength of 0.275 MPa and a drug delivery rate of 89.4% within 24 h [72]. Recently, an eco-friendly membrane based on chitosan and cellulose has been developed via aqueous phase separation. The membrane was highly porous 68.1% and mechanically resistant up to 15.5 bar under hydrodynamic pressurized conditions [73]. In this sense, a fibrous membrane composed of PLA and cellulose acetate was developed to enhance the mechanical properties of the PLA membrane. This composite membrane had a tensile strength of 9.3 MPa and a resistance to hydrostatic pressure of 16.5 KPa [74]. Similar improvements were developed for gelatin membranes using bacterial cellulose as filler. Pure gelatin membranes typically exhibit low mechanical strength and a poor vapour–water barrier; therefore, their applications are limited. The membrane developed had a tensile strength increased by 107% [75].

Biopolymer-based nanocomposites employ the same composite material principle, utilizing nanofillers to achieve their properties. The interaction between biopolymers and nanoparticles aims to enhance the biopolymers’ affinity, as well as their physicochemical properties, and improve their mechanical, thermal, and biological properties. The nanofillers could be spherical, rod-shaped, and needle-shaped; also, they could be classified as organic (e.g., cellulose, chitosan, and collagen), inorganic (e.g., cooper, silver, titanium oxide, zinc oxide, cerium oxide, aluminum oxide, copper oxide, and iron oxide), clay, and carbon-based (e.g., carbon nanotubes, graphene, and graphene oxide) [76]. Peter et al. (2024) developed a nanocomposite of calcium phosphate, nanocellulose, and chitosan [77]. The nanocomposite was operated at a high flux rate of 146 L/m^2^h MPa and had a pollutant removal rate of 98.7% for Nickel ions and 100% for Congo red dye.

Biopolymer-based blend nanocomposites are another alternative for enhancing membrane properties, utilizing a blend of biopolymers and nanofillers. The crucial role of nanoparticles is enabling a good molecular interaction between two biopolymers that are either compatible or naturally incompatible, thereby increasing their affinity. Additionally, they can alter the viscoelastic properties of the biopolymer blends [78]. For the development of PLA and polyhydroxybutyrate (PHB)-based blend films, cellulose nanocrystals were used; their presence in the blends improved the water vapour transmission rate (90.53 g/m^2^·day) [79]. A potential membrane for oil separation was prepared using a blend of PLA, polybutylene succinate, polypropylene carbonate, PHB biopolymers, and silica nanoparticles as fillers. The hydrophilic membrane had a suitable surface roughness, porosity, and thermal stability. The separation performance revealed oil removal from 17.127 mg/L to 0.582 mg/L [80]. Interestingly, the low water solubility and poor mechanical properties of chitosan were counteracted by blending it with the agar-agar biopolymer and using TiO_2_ nanoparticles as a cross-linking agent. The membrane had photocatalytic and antibacterial activities [81]. For tissue engineering applications, a nanocomposite material was composed of pectin-mediated hydroxyapatite, collagen, chitosan and magnesium oxide nanoparticles to stabilize the polymeric matrix. The membrane enhanced its mechanical strength, biocompatibility, and bioactivity, particularly in terms of cell adhesion [82].

Commercial production of biopolymer-based membranes remains constrained due to the high costs of biopolymer production compared with synthetic polymers. Polyhydroxyalkanoate polymers cost around $15,000/ton, polylactic acid polymers cost around $4000/ton, and synthetic polymers cost around $1000–$1500/ton [48]. Despite this, biopolymers have promising biodegradability, an advantage that can facilitate recycling and end-of-life disposal of the membranes. A further circular economy (reduce, reuse, and recycle) approach, utilizing biorefinery, biomass and waste valorization, engineering strategies, optimizing fermentation and processing, biotechnological tools, artificial intelligence, and optimal scale-up for biopolymer production, can significantly reduce prices [83]. Another limitation is the poor versatility, limited mechanical properties, insufficient thermal resistance, excessive swelling, a short lifespan in aqueous environments, chemical stability, poor film-forming capacity, and poor performance in industrial applications [15,84]. All these criteria remain as emergent research and optimization for a competitive biopolymer-based membrane, with the goal of being widely introduced to the market. The applications of biopolymer-based membranes are limited and are summarized in Table 1.

The real prominence of biopolymers in sustainable membrane manufacturing requires further research collaboration across the entire membrane production line to adopt an eco-friendly approach, optimize energy consumption, and achieve zero waste, non-toxicity, recyclability and biodegradability [85]. Biopolymers must deliver competitive functional membrane performance, equal to or better than commercial synthetic membranes in selectivity, permeability, and stability (e.g., CO_2_/CO selectivity range of 7–20 and up to 80 for H_2_CO_2_) [86,87]. Simultaneously, it must guarantee an almost zero environmental impact throughout its life cycle, from sourcing raw materials and the membrane preparation process to operational performance in harsh conditions and final disposal of the membrane. Moreover, it must guarantee scale-up potential and cost-effectiveness for both the commercial and industrial sectors. All of the above should be accompanied by economic, environmental and social policies that raise awareness of sustainability. Life cycle analysis is a powerful tool for revealing environmental and economic sustainability, using indicators such as marine ecotoxicity, freshwater eutrophication, ozone formation, carbon dioxide emissions, human carcinogenic toxicity, water consumption, and energy consumption. It is also possible to estimate production costs and environmental costs [88].

**Table 1:** Biopolymer-based membranes. ND: not determined.

Biopolymer	Method & Solvent	Membrane Properties	Application	Environmental Impact	Reference
Carboxymethyl chitin/nano-hydroxyapatite	Freeze dryer and cast & ethanol.	- Janus structure. The dry membrane was about 1 mm thick. Porous top layer of 100–200 μm, and dense bottom layer. - Water absorption of 13 g/g. Tensile stress–strain of 0.3 MPa. - Hemocompatibility (hemolysis rate < 5%). Cytocompatibility (cell viability > 92%) - Osteoconduction (MC3T3-E1 cell proliferation over 200% at 7 days in the porous layer). Barrier effect (no adhesion to NIH 3T3 fibroblasts in the dense layer). - Bone regeneration (about 10.03 ± 1.81% bone volume/total volume ratio).	Bone regeneration.	Biodegradability (over 60% after 27 days with 2 mg/L lysozyme).	[85]
Modified lignin/zeolite/chitosan	Casting/dichloromethane-dimethylformamide	- Composite, surface micropores. - Tensile strength between 10 and 25 MPa. Elongation at break between 5 and 10%. Water vapour permeability between 40 and 50 g/m^2^·24 h. Oxygen and carbon dioxide permeability between 1 and 3 (cm^3^(m^2^·24 h·0.1 MPa)). - Antioxidant activity (DPPH scavenging rate between 25 and 50%). - Antimicrobial activity (inhibition of *E.coli* growth between 20 and 40%).	Preservation of perishable foods.	No eco-friendly solvents were used.	[89]
Chitosan/pectin/UiO-66-NH_2_ nanoparticles	Casting & acetic acid, water	- Composite. - Maximum adsorption capacity of 168 mg/g for Arsenic(III). And, 335 mg/g for Arsenic(V).	Detoxifying arsenic from water.	- Water remediation. - Membrane reutilization during three cycles.	[90]
Polylactic acid-cellulose diacetate/Polylactic acid-carbon nanotubes	Layer-by-layer electrospinning & Dichloromethane and dimethylformamide.	- Asymmetric wettable Janus nanofiber. - Opposite wettability (hydrophobicity and hydrophilicity). - Tensile strength of 6.17 MPa with the addition of 0.5 wt% carbon nanotubes. - Decomposition temperature between 310.3 °C and 331.9 °C. - Separation efficiency over 89% for water-in-oil emulsions, and over 87% for oil-in-water emulsions.	Oil/water separation	- Water remediation. - No eco-friendly solvents were used.	[91]
Pullulan/chitosan/salvianolic acid	Centrifugal spinning/citric acid solution	- Fibrous (fiber diameter between 384 ± 123 and 644 ± 141 nm). - Cell inhibition rate against HCT116 colon cancer cells between 83.4% and 90.3%.	Drug delivery system	ND	[92]
Pectin/Ammonium iodide	Casting/water	- Amorphous. - Glass transition temperature of 38.6 °C for 70 M.wt% ammonium iodide. - Electrochemical stability at 2.71 V. - Ionic conductivity of 4.5 × 10^−3^ S cm^−1^ at room temperature.	Electrochemical application	- Proton battery for low energy density, and eco-friendly.	[93]
Collagen	Nanofibrillation and functionalization	- Nanofiber (dispersed nanofibrils of ~120 nm diameter). - Removal efficiency over 97% for PM_0.3_: 0.3 μm air particles. - Bacterial inactivation efficiency of 99% against *S. aureus* and *E. coli*.	Air purification	- Air remediation. - Bacterial inactivation efficiency over 50 reuse cycles. - Enzymatic degradability of 99% at 15 days using acid protease. Simulated landfill degradation of 50% after 63 days. - Life-cycle-analysis revealed the lowest impact in 16 of 18 environmental indicators.	[88]

## 6. Research Opportunities in Biopolymer Production for Membrane Manufacturing

While technological advances in membrane materials offer increasingly competitive and efficient alternatives, ecosystems, animal life, and human life are paying an ever-higher price. Therefore, the development of greener methods for biopolymer production, as well as increasing manufacturing cost-effectiveness, increasing global production volumes, and increasing the number of manufacturers, is a worldwide concern. Bioengineering tools present a promising avenue for producing biopolymers from plants [94], as well as from microorganisms such as bacteria [95], fungi [96], yeast, microalgae, and cyanobacteria. This process resembles a biological factory [97] with potential advantages, including minimal environmental pollution, profitability, high purity, and the ability to modify the biosynthetic pathways of biopolymers [98,99]. Extracellular microbial biopolymers, such as exopolysaccharides, extracellular proteoglycans, and other high-molecular-weight substances, are naturally produced by microorganisms as a defence mechanism against environmental stress. Pathogenic bacteria can also be utilized as a biosynthesis pathway for engineering and designing cell factories with the potential to synthesise novel biopolymers [18]. Novel exopolysaccharides were produced through the fermentation process under anaerobic conditions of *Enterococcus* species isolated from the vagina of pregnant women [100]. Cyanobacteria species have also shown biotechnological potential for polyhydroxybutyrate production, with evolutionary adaptations to stress conditions [101]. Another way to enhance the properties and activities of conventional biopolymers was described using biotechnologically produced chitosans, where enzymatic *N*-acetylation shows an advantage of directing acetylation patterns [102].

Environmentally, the strategic use of food and agro-industrial waste as a carbon source in fermentation bioprocesses for biopolymer production is currently a sustainable alternative [103]. A recent study demonstrated that microbial biorefinery technology and waste valorization offer a synergistic opportunity for producing extracellular biopolymers and intracellular lipids using *Rhodotorula* yeast [104]. Basri et al. (2025) developed nanocomposite films based on the principles of waste valorization and the circular economy [105]. They utilized extracellular polymeric substances secreted by the microbiome from a sewage treatment plant, chitosan, and calcium oxide nanoparticles derived from waste chicken eggshells. Furthermore, the membranes enhanced their thermal, optical, and mechanical properties, with a tensile strength increased by 16.2%, and an elongation at break of 186%. All these promising biotechnological applications for the production and modification of new extracellular and intracellular biopolymers can be enhanced by controlling various biotic and abiotic factors, as well as through genetic changes. Notably, several biopolymers, including nostoflan, cyanoflan, sacran, alternan, welan, levan, curdlan, kefiran, and mauran, remain largely unexplored for membrane applications [106].

Moreover, advances in biopolymer scale-up production are necessary to improve both upstream and downstream processes, optimize bioprocesses, and increase volumetric productivity; also, develop and design efficient modules for outdoor operations. Computing tools and novel technology can help to transfer laboratory operations to an industrial scale [107]. Furthermore, additional research is needed to develop homogeneous methodologies, system boundaries, and functional units for life cycle analysis based on ISO 14040:2006 standard [108]. This will enable faster comparisons of membrane manufacturing and more accurate decision-making [12]. It will be crucial to evaluate the sustainability of biopolymers within the framework of the circular economy. Additional awareness is needed, accompanied by government policies that promote research and business models through joint efforts by academic institutions, companies, government, and society, which can significantly lead to the replacement of conventional polymers with sustainable biopolymers. Policies are also necessary to promote the production, importation, commercialization, distribution, consumption, and waste management of biopolymers worldwide [109].

## 7. Conclusions

Sustainable membrane technology development is an emerging action toward an advanced future based on a circular economy framework, free from waste, environmental impact and human toxicity. To achieve this transition, significant scientific and technological efforts are required to discover eco-friendly materials, enhance their properties for high functional and operational performance, develop fouling-mitigation strategies, develop engineering procedures for large-scale applications, and conduct comprehensive studies on the principles of green membrane materials and processes. Biopolymers represent a promising option, although their use in membrane development is still in the early stages. Further research is needed to enhance the properties of existing biopolymers and to develop novel biopolymers with superior operational performance and competitive pricing. Additionally, arduous life-cycle analyses are necessary to make meaningful comparisons, improve energy efficiency, assess environmental impacts, and reduce costs. Such advancements could revolutionize green membrane technology and broaden its industrial-scale potential.

## Data Availability

No new data were created or analyzed in this study. Data sharing is not applicable to this article.
